# Stress Cardiovascular Magnetic Resonance Imaging for the Detection of Coronary Artery Disease

**DOI:** 10.31083/j.rcm2409254

**Published:** 2023-09-18

**Authors:** Giulia Vinco, Clementina Dugo, Marzia Rigolli, Cristina Demattè, Cristiana Giovanelli, Fabio Caruso, Alessandro Marinetti, Andrea Chiampan, Carmelo Cicciò, Carlo Cosimo Quattrocchi, Giulio Molon, Maurizio Del Greco

**Affiliations:** ^1^Division of Cardiology, Santa Maria del Carmine Hospital, APSS, 38068 Rovereto, Italy; ^2^Division of Cardiology, IRCCS Sacro Cuore Don Calabria Hospital, 37024 Negrar, Italy; ^3^Division of Cardiology, Department of Medicine, Azienda Ospedaliera Universitaria Integrata Verona, 37126 Verona, Italy; ^4^Division of Radiology, Santa Maria del Carmine Hospital, APSS, 38068 Rovereto, Italy; ^5^Division of Radiology, IRCCS Sacro Cuore Don Calabria Hospital, 37024 Negrar, Italy; ^6^Center for Medical Sciences - CISMed, University of Trento, 38122 Trento, Italy

**Keywords:** cardiovascular magnetic resonance imaging, myocardial perfusion, myocardial ischemia, coronary artery disease

## Abstract

Stress cardiovascular magnetic resonance (CMR) imaging has received extensive validation for the assessment of ischemic heart disease. Without ionizing radiation exposure, it offers in-depth information regarding cardiac structure and function, presence and degree of myocardial ischemia and myocardial viability. When compared to other imaging modalities, it has demonstrated excellent sensitivity and specificity in detecting functionally relevant coronary artery stenosis, as well as strong prognostic value in clinical risk stratification. The current scientific data support a greater expansion of stress CMR. This review investigates the current stress CMR techniques and protocols, as well as its relevance in diagnosis and prognosis of coronary artery disease.

## 1. Principles of Cardiovascular Magnetic Resonance

Cardiovascular magnetic resonance (CMR) imaging is a non-invasive and non-ionizing technique used to assess cardiovascular morphology, ventricular function, myocardial perfusion, tissue characterization and flow quantification. Images in CMR are based on the spatial and temporal reconstruction of amplified signal following the exposure of the hydrogen nuclei to high intensity static magnetic field, radiofrequency pulses and multidimensional magnetic field gradients. Once the patient is placed in the scanner static magnetic field, the hydrogen nuclei align along the magnetic field axis. Through the application of radiofrequency pulses, this baseline precession of the hydrogen nuclei can be perturbed. Perturbation signals are analyzed to derive a representation of the body’s protons’ spatial distribution [1]. This process provides images of the heart with a high anatomic resolution. CMR is thereby regarded as the gold standard imaging method for measuring biventricular volumes, mass, and function [2, 3]. In addition, CMR allows detailed myocardial tissue characterization, including assessment of edema, fat and fibrosis. Tissue contrast is mainly determined by the relaxation properties of hydrogen nuclei density, which varies between different tissues and is proportional to the water content. The two main relaxation times used in CMR are longitudinal relaxation, also called T1 relaxation, and transverse relaxation or T2 relaxation [4]. These two relaxations take various amounts of time in different tissues, since a different proportion of hydrogen nuclei characterize each tissue. Various radiofrequency pulse sequences are used in CMR, which allows to explore the heart composition. For example, T2-weighted pulse sequences are used to detect the presence of water in the myocardium, whereas T1-weighted images allow to detect fat infiltration and fibrosis [5, 6].

Gadolinium-based contrast agents (GBCAs) are administrated with an intravenous injection during CMR exams and tend to accumulate in areas of increased extracellular space, such as necrotic or scarred myocardium. Gadolinium has strong paramagnetic properties and decreases the T1 values of the surrounding tissue where it accumulates, resulting in enhancement on T1-weighted images, currently acquired 15–20 minutes after contrast intravenous administration (late gadolinium enhancement [LGE]). Based on specific and typical distribution patterns, LGE allows recognition of distinct cardiac diseases. Usually, LGE with an ischemic pattern follows coronary artery distribution and is typically subendocardial or transmural, whereas non-ischemic LGE patterns show a subepicardial or mid-wall distribution [7, 8, 9].

One of the most recent CMR tools available is parametric mapping. Differently from conventional imaging techniques, where signal intensity of pathological tissue is visually recognized as relative change compared to surrounding normal appearing tissue, parametric mapping provides a colour-coded representation of T1 and T2 times quantification pixel by pixel. T1 and T2 mapping values are expressed in units of time (e.g., milliseconds) and allow to infer tissue type and composition [10]. Direct quantitative mapping identifies diffuse disease by comparing relaxation time to previously determined normal range values and does not rely on visual assessment or semi-quantitative analysis as in LGE detection. In this regard, LGE conventional images are more appropriate in recognition of focal fibrosis (e.g., necrotic scar in ischemic cardiomyopathy), whereas parametric mapping is ideal to detect a diffuse myocardial disease (e.g., amyloidosis). Lastly, direct quantitative mapping allows tissue characterization without the necessity of exogenous contrast agents and therefore it can be used in patients with chronic kidney disease without increasing the risk of drug-related adverse events [11].

Unlike the scan of other structures, CMR faces the challenge of acquiring images of a moving organ, due to cardiac cycles and breathing movements. Thus, artifact-mitigated imaging in CMR relies on cardiac gating, which allows to acquire information during periods of relative cardiac quiescence, for example at end diastolic or end systolic phases [12, 13]. Moreover, the image acquisition is usually performed during short periods of end-expiratory apnea providing the patients with breathing instructions during the scan.

Before a CMR examination, the presence of absolute or relative contraindications must be carefully assessed. The static magnetic field and the radiofrequency pulses delivered during the examination may interfere with the correct function of metallic devices, such as pacemakers, implantable cardioverter defibrillators and loop recorders. Moreover, they may cause heating and damage to the surrounding tissue or cause device or lead displacement [14].

Nowadays, the majority of devices are magnetic resonance imaging (MRI) conditional, therefore patients can safely undergo the exam, as long as clinicians follow the manufacturer’s guidelines and wait the recommended amount of time after implantation, usually six weeks [15]. Abandoned or fractured pacemaker leads and epicardial leads are considered MRI unsafe and alternative imaging modalities should be considered instead of CMR [16]. Most of coronary and peripheral vascular stents are safe to be scanned just after the implantation, as the vessel wall prevents stent motion during the exam [17]. Finally, individuals with metallic objects in soft tissues like the brain, spine, or eyes should be thoroughly checked before the scan, to assess the risk of movement, torque and heating.

Regarding possible side effects, very subtle amounts of gadolinium, as the result of long-term retention into different organs and tissues, has been documented for patients with renal function impairment as well as with normal renal function. To date, no relationship of gadolinium retention with clinical consequences has been demonstrated and therefore gadolinium-based contrast agent administrations should be considered safe, while it should be used only when clinically indicated [18].

## 2. Stress CMR: How to Do it

### 2.1 Recommended Technique

Stress perfusion CMR is an accurate and non-invasive technique, which aims to detect myocardial ischemia [19]. Currently, clinical applications with stress CMR involve the use of pharmacologic agents, such as vasodilators (adenosine, regadenoson, dypiridamole) or dobutamine. Vasodilator stress perfusion testing is usually preferred and more commonly performed.

Exercise CMR with MRI-compatible treadmill or bicycle has been shown to be feasible, but it is not diffuse in clinical practice [20, 21]. Even if it provides a more physiological stress, it is affected by motion and breathing artifacts, impairing image quality, as well as limited availability due to the expensive equipment required.

Dobutamine stress CMR is usually employed when gadolinium is contraindicated or when contractile reserve needs to be assessed. Depending on the dosage, it can be used both to assess viability (at lower dose) or ischemia (at higher dose). Dobutamine increases myocardial contractility through beta-1 stimulation, therefore promoting myocardial ischemia by raising myocardial oxygen consumption in areas of reduced coronary perfusion, resulting in wall motion abnormalities [22]. The goal is to reach at least the 85% of the patient’s age adjusted maximum predicted heart rate (HR) response, using a graded dobutamine-atropine protocol. Although the use of gadolinium is not required, perfusion imaging can be added, increasing the test sensitivity and allowing for the detection of myocardial scars with LGE [23, 24]. The dobutamine stress CMR protocol includes the acquisition of three long-axis apical (2, 3, and 4-chamber) and at least three short-axis views at rest and at the end of each infusion increment [25]. To assess viability, dobutamine is infused at the dosage of 5 µg/kg/min for 3 min and then increased to 10 µg/kg/min for another 3 min. An improvement of at least one grade in wall motion at either the 5- or the 10- µg/kg/min dose is indicative of viability [26]. To assess myocardial ischemia, dobutamine is infused by increments of 10 µg/kg/min every 3 min, starting from 10 µg/kg/min until a maximum dose of 40 µg/kg/min. If the target HR is not reached, atropine can be administered in 0.5 mg incremental doses (up to a maximal dose of 2 mg). Images acquired during each stage of dobutamine infusion are therefore compared with baseline images to assess the development of new regional wall motion abnormalities, which indicate inducible ischemia. According to the 2019 European Society of Cardiology (ESC) guidelines, high event risk in patients with established chronic coronary syndromes is defined by at least 3 of 16 dobutamine-induced dysfunctional segments [27]. Once the stress protocol is completed, additional images are acquired to confirm that left ventricular (LV) wall motion has returned to baseline. Termination criteria include a new wall motion abnormality, achievement of the target HR, or a serious side effect. Patients should refrain from beta-blockers and nitrates for at least 12–24 h prior to the examination. Dobutamine stress CMR should not be performed in patients with severe systemic arterial hypertension (≥220/120 mmHg), unstable angina pectoris, severe aortic valve stenosis, complex cardiac arrhythmias including uncontrolled atrial fibrillation, hypertrophic obstructive cardiomyopathy, myocarditis, endocarditis, or pericarditis, uncontrolled heart failure. Atropine contraindications include narrow-angle glaucoma, myasthenia gravis, obstructive uropathy, obstructive gastrointestinal disorders [25].

**Fig. 1.**
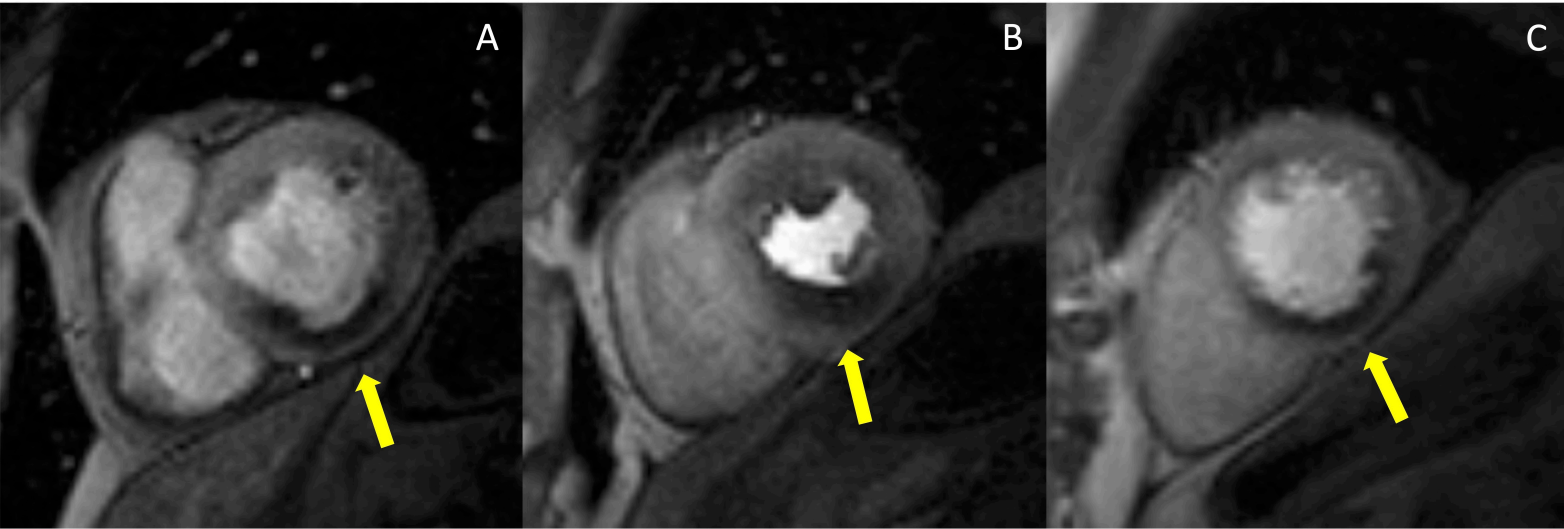
**Example of abnormal stress CMR**. The first-pass perfusion images are usually acquired in three short axis slices, at the basal (A), mid (B) and apical (C) ventricular level during coronary maximal vasodilation. This example shows an inducible perfusion defect, appearing as a hypointense subendocardial area (indicated by the yellow arrow lines) in the inferior and infero-septal segments. CMR, cardiovascular magnetic resonance.

Stress CMR with vasodilators induces a myocardial perfusion defect with the coronary steal phenomenon. During the administration of a vasodilator, the blood flow is increased in regions perfused by normal coronary arteries and reduced in areas supplied by stenotic arteries. Once hyperemia is obtained, serial dynamic stress perfusion images are continuously acquired during administration of intravenous contrast agent. Images are usually acquired in a stack of three short-axis planes covering the entire ventricle and an optional long axis plane. Care should be taken to avoid the inclusion of the LV outflow tract in the most basal short-axis slice. The post-contrast signal enhancement is visualized as the injected gadolinium-based contrast agent enters into cardiac chambers and perfuses the myocardium. In normally perfused myocardial segments, the contrast agent will enter faster and evenly, inducing a quick and higher increase in T1 signal intensity compared to areas supplied by stenotic arteries [28]. Hypoperfused areas will show a segmental subendocardial hypointense stripe (Fig. 1). According to the 2019 ESC guidelines, high event risk in patients with established chronic coronary syndromes is defined by at least 2 of 16 segments with stress perfusion defects [27].

**Fig. 2.**
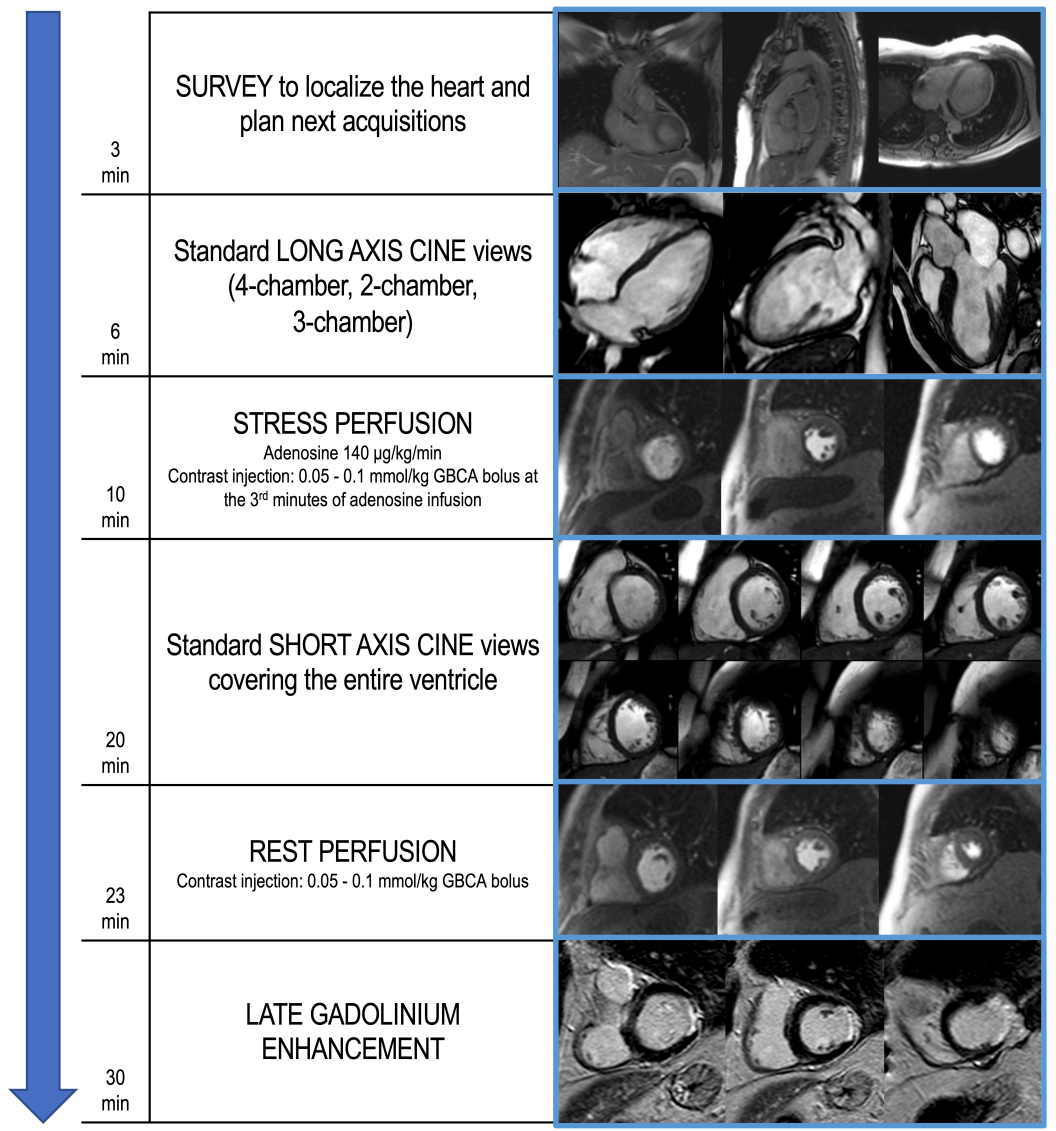
**Example of stress CMR protocol with adenosine**. The suggested protocol lasts about 30 min and starts with the acquisition of scout images to localize the heart (3 min). Standard long axis cine images are then acquired in 4-chamber, 2-chamber and 3-chamber orientation (6 min). First-pass perfusion images are therefore acquired in three short axis slices, during hyperemic conditions obtained with the administration of a vasoactive agent (i.e., adenosine), to assess for perfusion defects (10 min). Dosage of adenosine and contrast agent infusion are reported. In the following minutes, short axis cine images covering the entire ventricle are performed (20 min). At least 10 min after stress perfusion, rest perfusion images are acquired (23 min). About 5 min after the 2nd GBCA bolus injection, LGE images are performed, investigating the presence of myocardial scars (30 min). CMR, cardiovascular magnetic resonance; GBCA, gadolinium-based contrast agents; LGE, late gadolinium enhancement.

Among the vasodilators, adenosine is the most commonly used stress agent. The dose suggested is 140 µg/kg/min with an increase up to 210 µg/kg/min, if necessary, to achieve adequate stress [25]. The duration of adenosine infusion is standardised and usually lasts at least 3 min prior to contrast administration and data acquisition, with a total duration of the infusion of 4 min. Conventionally, adequate stress is defined by an HR increase of at least 10 bpm or a systolic blood pressure fall of more than 10 mmHg, since coronary vasodilatation is associated with systemic vasodilatation and reflex tachycardia. Once the stress perfusion image acquisitions are completed, the vasodilator is stopped (in the case of adenosine) or a pharmacologic agent is administered to reverse hyperemia (aminophylline when regadenoson or dipyridamole are used). To allow contrast wash-out from stress perfusion, a 10-minute pause is planned between stress and rest perfusion. Rest perfusion is then performed using the same parameters as for stress perfusion, but without the vasodilator administration. At least five minutes after the second contrast agent bolus has been administered, the sequences for LGE are acquired. LGE images will allow to identify the presence of ischemic scars and to assess myocardial viability. With this stress protocol the scanning time is about 30 min (Fig. 2).

**Fig. 3.**
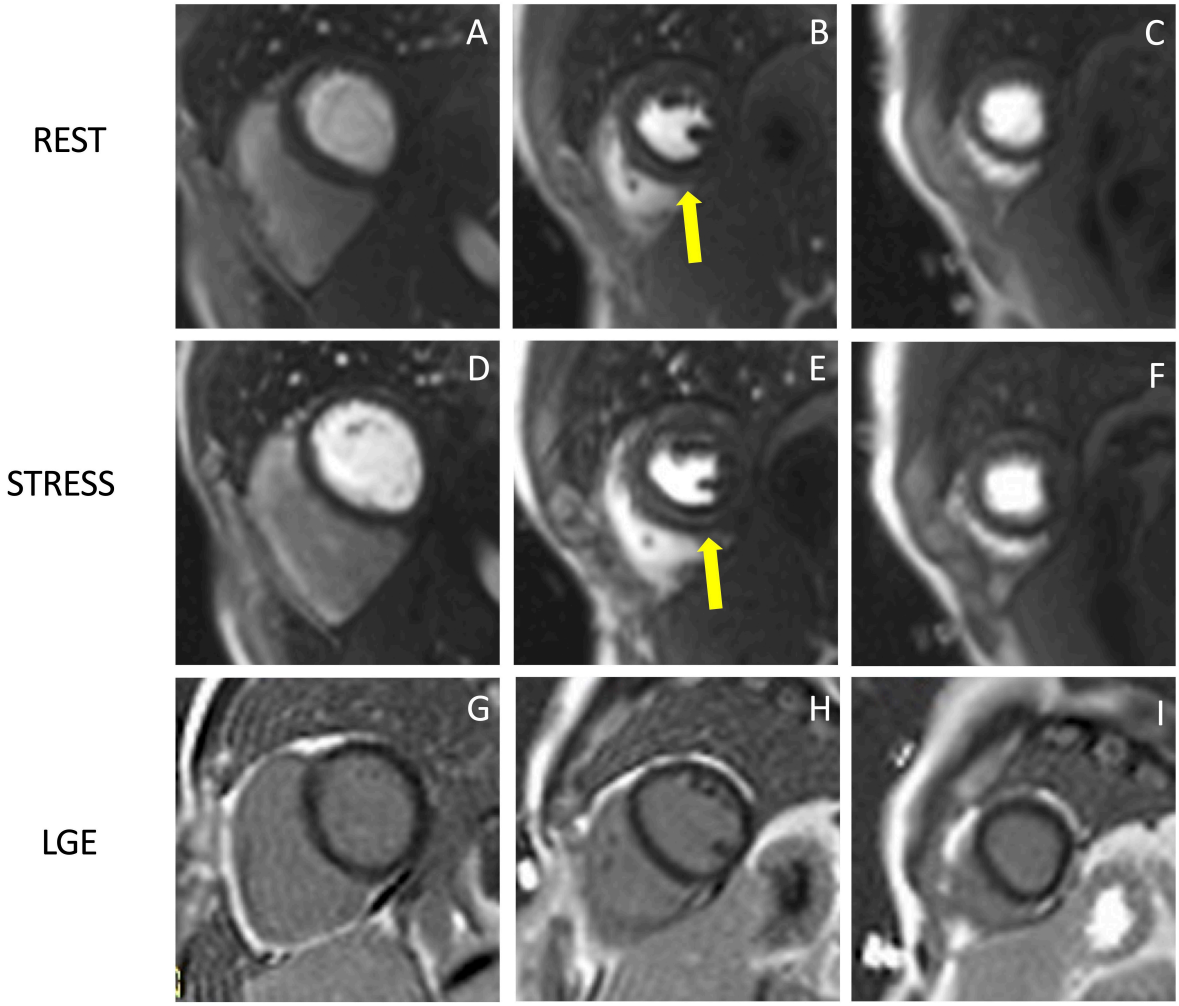
**Example of stress CMR images showing the ‘dark-rim artifact’**. CMR adenosine-stress perfusion in a 44-year-old man with a known congenital coronary artery abnormality (RCA with a high take off and inter-arterial course). Short axis rest and stress perfusion images are shown respectively at the basal (A,D), mid-ventricular (B,E), and apical (C,F) levels. There is evidence of a transient hypointense area in the subendocardial layer of the mid-ventricular septal segments (yellow arrows) both in the rest and stress images (B,E), during the early phase of passage of GBCA bolus through the left ventricle, suggestive for “dark rim artifact”. Corresponding LGE images (G,H,I) show no myocardial scars. CMR, cardiovascular magnetic resonance; LGE, late gadolinium enhancement; RCA, right coronary artery; GBCA, gadolinium-based contrast agent.

The perfusion images are analyzed using the American Heart Association (AHA) 16-segment model [29]. By assessing the number of segments with perfusion defects due to ischemia, the global ischemic burden can be estimated. The perfusion defect is usually most evident about three heart beats after the maximal contrast enhancement of the ventricular cavity and continues while the contrast washes out. It is important to distinguish inducible perfusion defects from artifacts. The most common is the ‘dark-rim artifact’, which appears as a hypointense area in the subendocardial layer of the myocardium, usually due to low spatial and temporal resolution (Fig. 3). Dark rim artifacts usually appear as the gadolinium first reaches the ventricular cavity, but it vanishes once the myocardium is enhanced. Moreover, they are present during both rest and stress conditions in the absence of underlying scars in the LGE images.

### 2.2 Safety Profile

Table 1 illustrates differences in commonly used vasodilators. Termination criteria of vasodilator stress testing include severe respiratory difficulty, persistent or symptomatic atrio-ventricular block, persistent hypotension or a significant drop in systolic pressure (>20 mmHg). Due to potential of interaction with the stress agents, patients should refrain from caffeine, theophylline, dipyridamole and nicotine for 12–24 h prior to the examination. Contraindications to vasodilators include: 2nd degree (type 2) or complete atrioventricular block, systolic blood pressure <90 mmHg, severe systemic arterial hypertension (>220/120 mmHg), sinus bradycardia (HR <40 bpm), active bronchoconstrictive or bronchospastic disease with regular use of inhalers, known hypersensitivity to stress agents [25]. Regadenoson has been shown to be a safer option in patients with mild-to-moderate chronic obstructive pulmonary disease and asthma compared to other vasodilators and can therefore be considered in these cases [30, 31, 32].

**Table 1.** **Commonly used vasodilators in stress CMR**.

	Adenosine	Regadenoson	Dypiridamole
Dose	140 µg/kg/min	0.4 mg	0.142 µg/kg/min
Dose adjustment	up to 210 µg/kg/m*	/	/
Duration of infusion	4 min	single injection (10 s)	4 min
Half life	<10 s	${\mathrm{triphasic}}^{†}$	10 h
IV accesses	2 (one for each arm)	1	2 (one for each arm)
Timing for GBCA injection	at the 3rd min of infusion	1 min after injection	2 min after injection
Reversal agent	/	aminophylline 100 mg IV	aminophylline 100 mg IV

* if HR does not increase by 10 bpm or SBP does not drop by >10 mmHg. ^†^ Initial phase: 2 to 4 min; intermediate phase: 30 min; terminal phase: 2 h. CMR, cardiovascular magnetic resonance; GBCA, gadolinium-based contrast agents; IV, intravenous; SBP, systolic blood pressure.

**Fig. 4.**
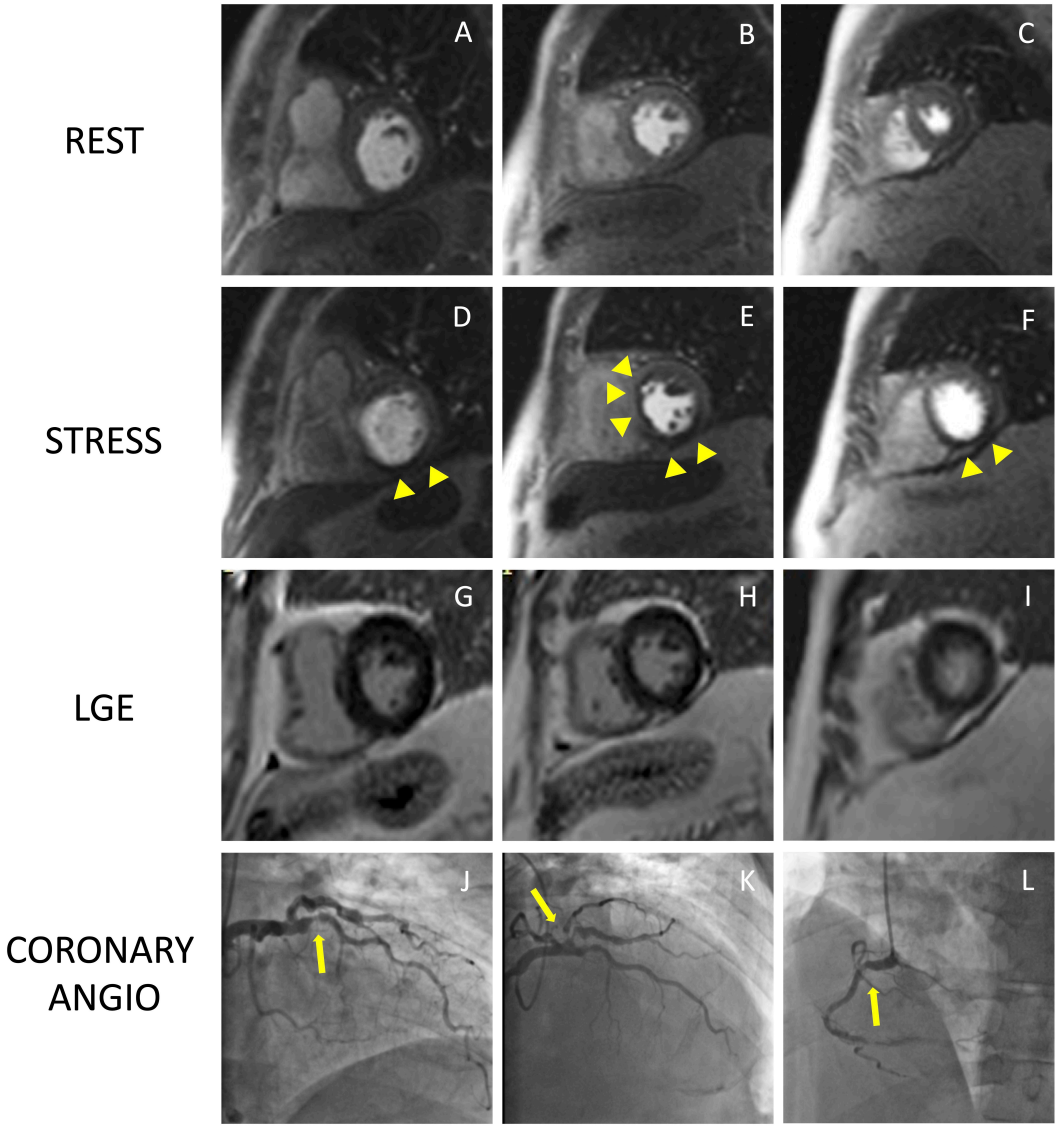
**Example of positive CMR adenosine-stress perfusion**. We present the case of a 73-year-old man with new onset of ventricular arrythmia on exercise test and a history of previous ACS and RCA angioplasty. Short axis rest and stress perfusion images are shown respectively at the basal (A,D), mid-ventricular (B,E), and apical (C,F) level. The stress images show the presence of a perfusion defect, appearing as subendocardial hypointense area in the inferior septum, inferior wall and in the mid portion of the anterior and antero-septal walls (yellow arrow heads). Corresponding LGE images (G,H,I) show no myocardial scarring. The patient underwent a coronary angiography which revealed diffuse CAD with severe stenosis at the proximal tract of the LAD artery (yellow arrow line, J). and at the origin of the intermediate and the first diagonal branches (yellow arrow line, K). Moreover, there was an intrastent occlusion in the RCA with a collateral circulation (yellow arrow line, L). CMR, cardiovascular magnetic resonance; ACS, acute coronary syndrome; CAD, coronary artery disease; LAD, left anterior descending; LGE, late gadolinium enhancement; RCA, right coronary artery.

**Fig. 5.**
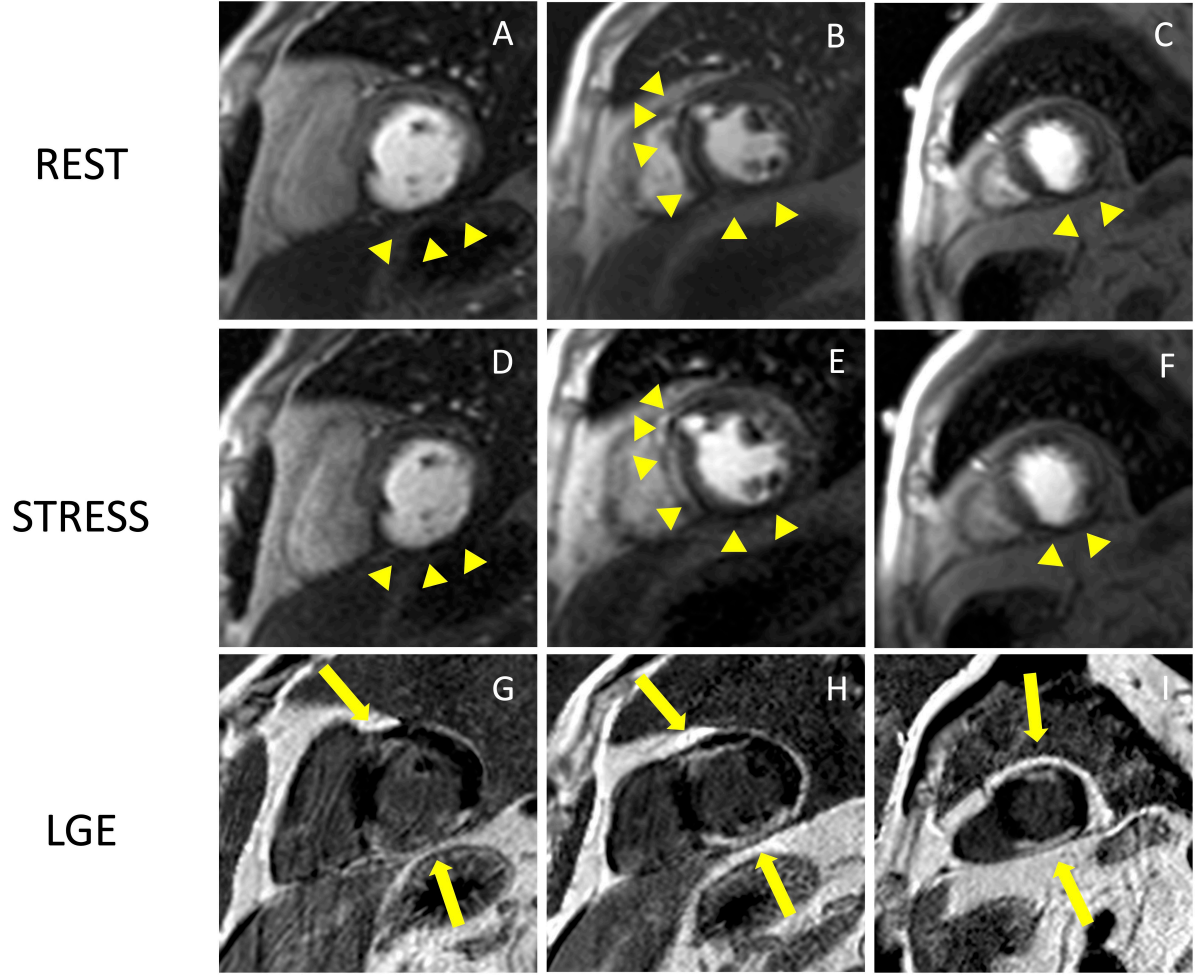
**Example of CMR adenosine-stress perfusion in the presence of ischemic scar**. This is the case of a 68-year-old man with a history of subacute myocardial infarction and previous unsuccessful percutaneous angioplasty on the RCA. Short axis rest and stress perfusion images are shown respectively at the basal (A,D), mid-ventricular (B,E), and apical (C,F) level. There is evidence of hypoperfusion, appearing as a hypointense subendocardial area in the inferior septum, inferior wall and in the mid portion of the anterior wall (yellow arrow heads). Corresponding LGE images (G,H,I) show ischemic scars (yellow arrow lines) with a transmural distribution in the inferior septum and inferior wall. Moreover, there is subendocardial LGE with a 50–75% transmurality in the anterior wall. The perfusion defects appear both in the rest and stress images and are related to the presence of non-viable myocardium (scar transmurality >50%), as shown in the LGE images. The patient was deferred from coronary revascularization. CMR, cardiovascular magnetic resonance; LGE, late gadolinium enhancement; RCA, right coronary artery.

**Fig. 6.**
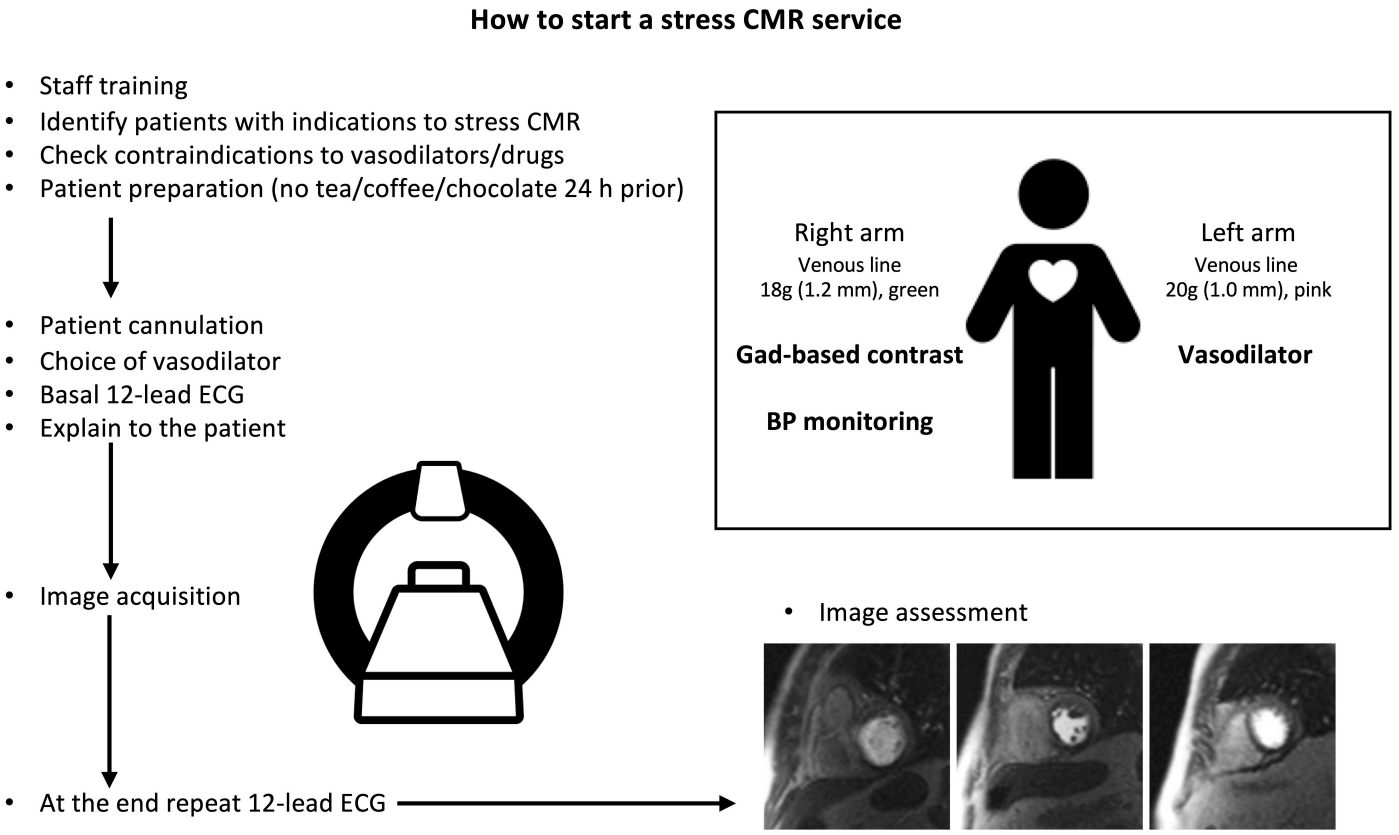
**How to start a stress CMR service**. This image shows all the practical steps necessary to implement a stress CMR service in the clinical practice and optimize the workflow. CMR, cardiovascular magnetic resonance; BP, blood pressure; ECG, electrocardiogram; Gad, gadolinium.

Stress CMR has been shown to be a safe and feasible technique, with good diagnostic quality. In a multicentre prospective registry of about 12,000 referral patients, stress CMR showed an excellent safety profile with higher incidence of mild complications and minor symptoms in dobutamine stress CMR compared to other vasodilators [33]. The safety of stress CMR has been demonstrated in patients with heart failure with reduced ejection fraction without any adverse event in a population of 1053 patients [34]. Stress CMR was well tolerated also in consecutive patients with MR-conditional pacemakers, with no significant change in lead thresholds or pacing parameters [35]. Pezel *et al*. [36] demonstrated the feasibility of vasodilator stress CMR in more than 600 patients with atrial fibrillation and suspected or stable coronary artery disease (CAD). Fig. 4 and Fig. 5 show two examples of stress CMR clinical cases. Fig. 6 shows how to implement stress CMR in clinical practice.

## 3. Diagnostic Accuracy of Stress CMR 

Growing evidence from numerous studies and meta-analyses demonstrates that non-invasive stress testing has a high diagnostic performance in identifying significant CAD when compared to techniques regarded as the gold standard, like invasive coronary angiography (CA) with fractional flow reserve (FFR) [37]. Due to its great sensitivity and specificity, stress CMR has been supported by numerous trials as an accurate method to assess patients with known or suspected CAD [38, 39]. Stress CMR resulted equivalent or superior to single-photon emission computed tomography (SPECT) in studies assessing diagnostic accuracy [40].

In 2008, a head-to-head comparison of adenosine stress CMR and SPECT with CA as the gold standard reported equal performance in the multicenter, multivendor, randomized trial MR-IMPACT (Magnetic Resonance Imaging for Myocardial Perfusion Assessment in Coronary Artery Disease Trial). Furthermore, stress CMR diagnostic performance was found to be superior to SPECT when comparing the entire study population (area under the receiver operating characteristics ROC curve (AUC): 0.86 ± 0.06 *vs.* 0.67 ± 0:05; *p* = 0.013) and especially in multi-vessel CAD (AUC 0.89 ± 0.06 *vs.* 0.70 ± 0.5; *p* = 0.006) [40].

Further evidence derived from the MR-IMPACT II (Magnetic Resonance Imaging for Myocardial Perfusion Assessment in Coronary Artery Disease Trial) in 2013, a large trial involving 533 patients in 33 centres, focused on evaluating the diagnostic efficacy of stress CMR and SPECT for the identification of CAD, which was defined as a reduction of at least 50% in the diameter of the coronary vessel in the CA. Stress CMR, compared to SPECT, resulted more sensitive (0.69 *vs.* 0.59, *p *= 0.024) but less specific (0.61 and 0.72, *p *= 0.038) for the detection of CAD [41]. In addition, the diagnostic performance of stress CMR was superior in several sub-groups analysis, such as in subjects with multi-vessel disease, in both male and female patients and in non-infarcted individuals [42].

The Clinical Evaluation of Magnetic Resonance Imaging in Coronary Heart Disease (CE-MARC) trial was a large prospective multicenter study that enrolled 752 patients with suspected angina pectoris and at least one significant cardiovascular risk factor who underwent stress CMR, SPECT, and CA, the latter of which served as the gold standard. Compared with SPECT, stress CMR had greater sensitivity, similar specificity and overall better diagnostic performance (AUC 0.89 *vs.* 0.74; *p* < 0.0001) in detecting severe CAD. In detail, stress CMR allowed an accurate assessment of both single-vessel and multi-vessel CAD, regardless of the cut-off value used to define significant vessel stenosis (≥50% or ≥70%). Of note, unlike previous studies concentrating on inducible ischemia only, the CMR protocol in CE-MARC included LV function, viability assessment and three-dimensional (3D) whole heart MR coronary angiography in addition to perfusion images. However, the addiction of coronary artery imaging to the common protocol did not result in improved diagnostic performance [19].

In a large meta-analysis comparing cardiac imaging techniques to invasive FFR (gold standard), stress CMR proved to have the highest diagnostic performance in detecting myocardial ischemia on both a per-patient (sensitivity = 90%, specificity = 94%) and per-vessel basis (sensitivity = 91%, specificity = 85%), whereas both SPECT and stress echocardiography (SE) had lower performance. Coronary computed tomography angiography (CCTA) and computed tomography-derived fractional flow reserve (FFR-CT) yielded lower specificity, with functional assessment of CAD by SE, SPECT and FFR-CT improving accuracy [37].

Finally, studies comparing stress CMR with invasive techniques such as CA and invasive FFR, reported high correlation between the techniques. Using both per-coronary territory and per-patient analyses, Watkins *et al*. [38] demonstrated that stress CMR provides high positive and negative predictive values (91% and 94% respectively) for diagnosing severe CAD as defined by invasive FFR. A stress CMR-based strategy was not inferior to FFR with regard to major adverse cardiac events in patients with stable angina and risk factors for CAD, but was associated with a lower incidence of coronary revascularization than invasive angiography with assessment of FFR (35.7% of patients *vs.* 45.0%, respectively) [39].

Given the present availability of numerous non-invasive techniques for identifying CAD, these findings are especially relevant and must be taken into consideration when selecting the optimal test to determine which patients would benefit from being referred for more invasive treatments. Stress CMR acts as a gatekeeper for CA and percutaneous coronary intervention (PCI) and is crucial in the functional assessment of individuals with known or suspected CAD. Moreover, it must be acknowledged that CMR has some benefits over other imaging modalities, such as excellent spatial and temporal resolution that enable volumetric analysis and tissue characterisation without exposure to ionizing radiation [27].

## 4. Prognostic Utility of Stress CMR 

Several studies and patient subgroups have assessed the prognostic value of stress CMR. LGE and inducible ischemia have both been associated with unfavorable outcomes.

In the Euro-CMR registry analysis, the authors reported a high negative predictive value of stress CMR in patients with suspected CAD and an event rate for cardiac death and non-fatal myocardial infarction (MI) as low as 1% per year [43]. More recently, in a large multicentre cohort of 2349 patients in the U.S. with stable chest pain, stress CMR showed excellent ability to predict the risk of cardiac events. Patients with no inducible ischemia and without evidence of LGE showed low annual rate of primary outcomes, defined as cardiovascular death or non-fatal MI, with an associated event free rate of 99.3% per year, over an intermediate follow-up period of 5.5 years. Conversely, patients with ischemia and LGE experienced primary outcomes with an annual rate of 4.5% and 10.1% respectively [44].

Another multicenter study examined 9151 patients who underwent stress CMR and had known or suspected CAD: those who had a positive perfusion stress exam had significantly higher yearly mortality than those who had a negative test. Also, researchers noticed a considerable improvement in the ability to predict negative occurrences when stress CMR was added as a variable in the Cox-regression analysis [45].

Thanks to LGE, CMR can visualize the presence of myocardial scarring resulting from ischemic events. The extent of LGE in terms of trasmurality allows to predict in a stepwise manner the likelihood of myocardial contractility recovery after coronary revascularization [46]. Moreover, the presence of LGE is known to be a predictor of adverse outcomes [47, 48]. In a cohort of older individuals, the prevalence of unrecognized MI by CMR was 17% and it was associated with an increased mortality risk [49].

The ISCHEMIA (International Study of Comparative Health Effectiveness with Medical and Invasive Approaches) trial [50] highlighted the role of optimal medical therapy in patients with stable ischemic disease and supported the use of non-invasive stress imaging modalities for the effective risk assessment to guide the use of invasive procedures in high risk patients.

Stress CMR has demonstrated meaningful improvement in risk reclassification in patients assessed for possible ischemic heart disease, especially in patients with moderate pre-test risk. Reclassification of 65.7% of patients to low risk and 25.8% of patients to high risk by inducible ischemia, with a low (0.3%) and a high (4.9%) annual risk of cardiac death and MI, has been demonstrated in a cohort of 815 consecutive patients referred for evaluation of myocardial ischemia [51].

The extent of ischemia and the threshold to consider it relevant to suggest coronary revascularization, is a topic of great interest. Data from an observational registry reported an increasing survival benefit with revascularization over optimal medical therapy in the setting of extensive ischemia (>10% of myocardium) both among patients without prior CAD and those with no prior MI [52]. In a large cohort of patients with known or suspected CAD referred to stress CMR, an ischemia burden of 1.5 or more segments was correlated with high rate of cardiac death, non-fatal MI and late coronary revascularization. Patients with ischemia below this threshold can be defined as “low risk” population and can be safely deferred from revascularization [47].

Finally, stress CMR may play a pivotal role in some subsets of challenging patients that are not suitable to evaluation with other non-invasive imaging modalities, such as women [53] and obese patients [54] in whom ultrasound acoustic windows are frequently poor resulting in non-diagnostic exams. Moreover, stress CMR may be particularly indicated in patients with possible silent ischemia, balanced ischemia and micro-vascular dysfunction such as diabetics [55] and in patients with reduced LV systolic function. In this latter subset, the technique proved a strong prognostic utility and demonstrated incremental value to the clinical model in predicting the primary outcomes [56].

## 5. Challenges and Limitations 

Although stress CMR is an excellent diagnostic tool for assessment of CAD, some limitations have to be acknowledged that might limit its widespread use. A major challenge of stress CMR is its limited accessibility related to the time-consuming nature and high costs. However, several studies have demonstrated its cost-effectiveness as a gatekeeping tool in patients at risk for obstructive CAD [57]. Some patients are claustrophobic and may experience fear of tight spaces, but this accounts for less than 2% of prematurely terminated scans [58]. CMR images in patients with implantable devices might be hampered by device-related imaging artifacts, limiting the interpretability of the images. The use of macrocyclic GBCAs in the recent years has substantially reduced concerns related to the risk of nephrogenic systemic fibrosis in patients with severe renal disease. Another challenge of stress CMR is given by the limited LV coverage of the three short axis slices acquired, possibly increasing false negatives in clinical practice [59].

## 6. Future Perspectives

T1 mapping techniques appear a promising tool in the assessment of patients with stable CAD, without the need for contrast agent administration. By acquiring native T1 mapping both at rest and during hyperemic conditions, T1 reactivity (percentage difference in T1 values between rest and stress) can be calculated. Recent studies on stress T1 mapping have shown a significant difference in T1 reactivity between normal and ischemic (or infarcted) myocardium [60, 61]. Studies showed no significant difference in T1 reactivity between ischemic and infarcted myocardium, but it was suggested that the infarcted myocardium can be identified by means of T1 values at rest. Future studies on larger populations should assess the diagnostic accuracy of native T1 and T1-reactivity for the detection of myocardial inducible ischemia.

Another important advance in stress CMR is the application of fully automated quantitative perfusion CMR techniques, generating pixel-wise perfusion maps [62]. Fully quantitative stress CMR showed a similar diagnostic accuracy in detecting CAD as conventional qualitative methods [63, 64]. Moreover, automated quantitative CMR perfusion mapping showed good diagnostic accuracy in detecting microvascular dysfunction and multivessel CAD [65]. The implementation of artificial intelligence and deep learning models to quantitative stress perfusion appears promising to obtain faster and accurate results compared to manual processing [66]. Artificial intelligence might be useful also to improve patient risk stratification: recently the application of a machine learning score considering both clinical and stress CMR data showed a higher prognostic value compared to traditional clinical or CMR scores [67].

## 7. Conclusions

Stress CMR has been widely validated for the evaluation of ischemic heart disease. Without the use of ionizing radiation exposure, it allows to detect inducible myocardial ischemia and myocardial viability and to assess global and regional ventricular function. CMR showed high sensitivity and specificity when compared with the gold standard of coronary angiography with FFR, as well as high prognostic relevance in clinical risk stratification. Despite a large body of evidence regarding its diagnostic and prognostic role as well as cost-effectiveness, this imaging modality is currently underutilized. More efforts should be made in the future to remove barriers to the widespread use of stress CMR for the evaluation of ischemic heart disease.
